# Development and psychometric evaluation of a lifestyle adherence assessment scale for patients with dry eye syndrome

**DOI:** 10.1186/s12886-025-04578-8

**Published:** 2026-01-09

**Authors:** Muling Li, Peiyan Zhu, Qinghui Huang, Muqing Wu, Chunmei Li, Yunji Wang, Jianpeng Wu, Juan Yang

**Affiliations:** 1 Department of Ophthalmology, Nanfang Hospital, Southern Medical University, No.1838 North of Guangzhou Avenue, Baiyun District, Guangzhou City, Guangdong Province PR China; 2 Department of Neurosurgery, Nanfang Hospital, Southern Medical University, No.1838 North of Guangzhou Avenue, Baiyun District, Guangzhou City, Guangdong Province PR China; 3 Research & Development Center, Guangzhou Inkee Technology Co., Ltd, 2339K, 23rd Floor, No.179, Tianhe North Road, Tianhe District, Guangzhou City, Guangdong Province China

**Keywords:** Dry eye syndrome, Lifestyle, Adherence, Nursing, Scale, Validity, Reliability

## Abstract

**Objectives:**

To develop the lifestyle adherence assessment scale for patients with dry eye syndrome and conduct validity and reliability analyses.

**Design:**

A cross-sectional and methodological scale validation design, following classical test theory.

**Methods:**

The study was conducted in three stages. The initial stage focused on item generation and revision, which involved a comprehensive literature analysis and expert inquiries. Phase two involved the assessment and exploration of items. The third phase involved a cross-sectional survey to establish construct validity, content validity, internal consistency reliability, and exploratory factor analysis. A total of 422 samples that met the study criteria were included during the second and third stages (June 2024 to March 2025).

**Results:**

The final scale contains 18 items across five dimensions: ophthalmic care standards, ocular strain regulation standards, seeking supports, tobacco and alcohol consumption regulations, and daily conduct guidelines. Twenty-three experts with professional experience participated in two rounds of expert consultations. Expert authority coefficients were 0.854 and 0.894 in two Delphi rounds, with 100% response and validity rates. Exploratory factor analysis extracted five factors accounting for 54.65% of the variance. Confirmatory factor analysis indicated good model fit (*χ²*/df = 3.580, CFI = 0.958, TLI = 0.947, SRMR = 0.072, RMSEA = 0.038). The scale-level and item-level content validity indices were both 1.000. Cronbach’s α was 0.894, and test-retest reliability was 0.931.

**Conclusion:**

The Lifestyle Adherence Assessment Scale for Dry Eye Patients has excellent reliability and validity, making it a suitable instrument for evaluating patients’ adherence to lifestyle modifications.

## Introduction

Dry eye syndrome, also referred to as keratoconjunctivitis sicca or ocular surface desiccation, is a chronic multifactorial disorder characterized primarily by tear film instability or an imbalance in the ocular surface microenvironment. As one of the most prevalent ocular surface diseases, it manifests as symptoms of ocular irritation (including conjunctival hyperaemia, ocular dryness, burning sensation, and foreign body sensation), reduced visual acuity, increased blink frequency, and meibomian gland dysfunction with obstruction [1]. The global prevalence of dry eye disease ranges from 5% to 50% [2], with China reporting a prevalence rate of 57.6% [3], which demonstrates a concerning trend among younger age groups. Currently, there is no definitive cure for this condition, and therapeutic interventions focus primarily on symptom management [4]. Untreated cases may progress to severe complications, including corneal ulcers, perforation, secondary infections, and neovascularization, potentially leading to significant visual impairment and, in severe cases, complete vision loss [5]. Compared with healthy individuals, patients with mild dry eye syndrome experience significant limitations in performing visually demanding tasks such as reading and driving. Furthermore, severe dry eye-induced blindness not only renders individuals incapable of participating in the workforce but also imposes substantial economic burdens on their families [6]. Consequently, the prevalence of anxiety and depression among dry eye patients is markedly greater than it is among healthy individuals, which adversely affects patients’ quality of life and psychological well-being [7].

“Lifestyle” is defined as the aggregate of daily behavior patterns shaped by socio-economic and cultural contexts, encompassing facets such as diet, attire, habitation, and mobility. These daily behaviours and habits have a significant influence on human health, including ocular surface health. Extensive research has demonstrated that the ocular surface is particularly susceptible to the influence of external environmental factors, including lifestyle and societal challenges, environmental conditions, digital environments, and the use of contact lenses and cosmetics, as well as internal factors, such as nutrition, selective medications, and surgical interventions. These factors collectively contribute to the onset of dry eye disease, which is increasingly recognized as a lifestyle-associated condition. The work “Chinese Expert Consensus on Dry Eye: Lifestyle-related Dry Eye (2020)” [8] classified lifestyle-related dry eye as a separate category, indicating that lifestyle is closely related to dry eye. It is highly important to assist patients in adopting a healthy, evidence-based lifestyle for the prevention and treatment of dry eye, which needs urgent attention and management. In our preliminary research, the team developed the " Best evidence summary for prevention and management of lifestyle-related dry eye” based on evidence-based theory [9], which serves as a reference for nursing professionals in clinical practice.

Assessing lifestyle adherence is a prerequisite for implementing effective measures to ensure long-term lifestyle management in patients with dry eye disease, and lifestyle adherence plays a predictive role in disease progression. Currently, there is a lack of validated assessment tools that are specifically designed to evaluate lifestyle adherence in dry eye patients. Previous research has relied predominantly on simplistic, self-developed questionnaires to assess individual lifestyle aspects, which has resulted in unidimensional evaluations that lack objectivity and scientific rigor [10]. Therefore, it is imperative to develop a lifestyle adherence scale for dry eye patients based on theoretical guidance and to validate its reliability and validity, thereby providing an effective tool for assessing lifestyle adherence in this patient population. In 1992, the World Health Organization synthesized global achievements in preventive medicine and introduced the “Four Pillars of Health” within the Victoria Declaration: balanced diet, moderate exercise, smoking cessation and alcohol restriction, and psychological balance. These four pillars have consistently been regarded as the most scientifically validated lifestyle principles. Consequently, this study, based on the “Four Pillars of Health” theory and previous evidence-based achievements, aims to develop a lifestyle adherence assessment scale that suits China’s national conditions. To obtain a comprehensive understanding of lifestyle adherence among dry eye patients, this study seeks to formulate improvement strategies to improve patients’ management of their lifestyle and to optimize disease treatment outcomes.

## Theoretical framework

**Fig. 1:**
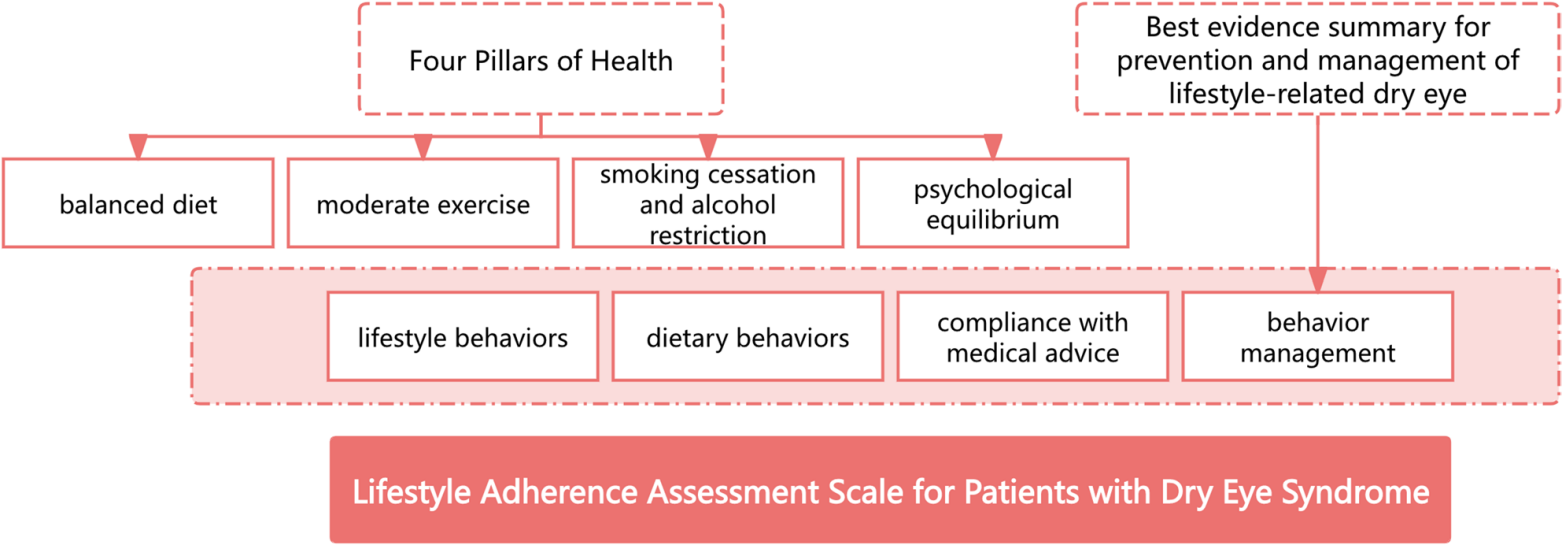
The theoretical framework for the Lifestyle Adherence Assessment Scale for patients with dry eye syndrome

The theoretical framework facilitates the interpretation and prediction of individuals’ behavioral preferences. This study is guided by the “Four Pillars of Health” framework proposed by the World Health Organization (WHO) [11]and the “Best evidence summary for prevention and management of lifestyle - related dry eye” previously developed by our team [9]. The “Four Pillars of Health” put forward by the WHO encompass four aspects: balanced nutrition, moderate exercise, smoking cessation and alcohol restriction, and mental health. The evidence - based summary ultimately aggregates five aspects of content related to lifestyle, including the management of artificial tears, eye care, diet management, health guidance, and follow - up education. Therefore, based on existing research and the two theories, this study has preliminarily constructed the dimensions of the lifestyle adherence assessment scale for patients with dry eye syndrome, which include four dimensions: living behaviour, dietary behaviour, adherence with medical advice, and behaviour management. The theoretical framework of this study is illustrated in Fig. 1.

## Methods

### Aims

The present study aimed to develop the lifestyle adherence assessment scale for patients with dry eye syndrome.

### Design

**Fig. 2:**
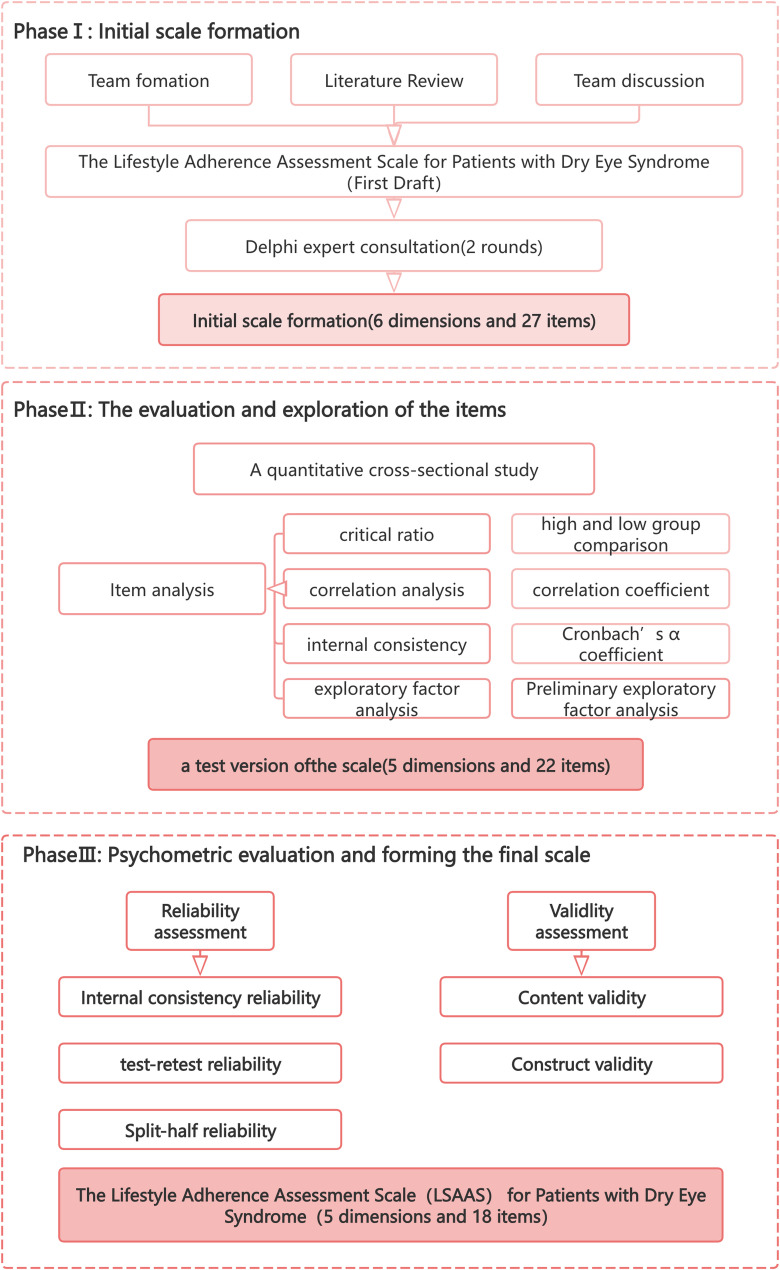
Development and Validation of the Lifestyle Adherence Assessment Scale for Patients​ with Dry Eye Syndrome

From June 2024 to March 2025, we conducted a multiphase study using both quantitative and qualitative approaches to develop and validate the Dry Eye Patient Lifestyle Adherence Scale, which involved three phases: (a) Initial Scale Formation; (b) item evaluation and exploration; and (c) psychometric evaluation of the scale. The scale development process is depicted in Fig. 2.

### Initial scale formation

#### Research team formation

A research group was established for this study, including one head nurse in ophthalmology, two master’s degree holders in nursing, four advanced practice nurses in ophthalmology nursing and one information technology professional.

#### Literature review

In PubMed, Embase, Web of Science, CINAHL, CNKI, China Wanfang databases, Joanna Briggs Institute, BMJ Best Practice, UpToDate, The Cochrane Library and etc, we conducted literature searches using keywords such as ‘dry eye syndromes/dry eye syndrome/dry eye disease*/dry eye*/evaporative dry eye disease/evaporative dry eye syndrome/ evaporative dry eye’、‘prevention’、‘nursing’ 、‘management’ 、‘treatment’ and ‘education’. Ultimately, based on the evidence-based medical approach, we have compiled the best evidence summary for the prevention and management of lifestyle issues in patients with dry eye [9].

Based on the literature review, this study used the brainstorming method to convene a group discussion within the subject group. Through discussions on the lifestyle adherence content of dry eye patients, group members put forward opinions or suggestions regarding each item and further revised and improved the item library. Finally, 4 dimensions and 37 items were formed and then the first round of the expert consultation questionnaire was organised.

#### Delphi method

This study implemented two rounds of expert consultation via email from June to August 2024. Twenty-three experts were finally selected for consultation in this study, with expert units located in Guangzhou, Zhuhai, Shantou, Huizhou and Shenzhen in China. Included criteria for experts were: ① nurses with a bachelor’s degree or higher in nursing and physicians with a master’s degree or higher; ② healthcare professionals with more than 5 years of experience in clinical ophthalmology, ophthalmic nursing, or chronic disease management; ③ nurses holding a supervisory or higher technical title and physicians holding an attending or higher technical title; and ④ informed consent and voluntary participation. The exclusion criterion was failure to provide timely feedback on the consultation questionnaire.

The expert consultation questionnaire consisted of three parts: the consultation instructions, the main body of the questionnaire, and the basic information of the experts. Each expert used a 5-point Likert scale (“very unimportant” was given a score of 1 point, “very important” was given a score of 5 points) to evaluate the relevance of the scale items to the theme and provided comments. Based on the importance ratings of the items and the experts’ suggestions, items were added, removed, or modified. Items with an importance score < 3.50 or a coefficient of variation > 0.25 were excluded [12]. The questionnaire response rate was used to assess the experts’ engagement level. The authority coefficient represents the mean value of domain familiarity and indicator evaluation criteria. The degree of consensus among experts was determined by Kendall’s coefficient of concordance, which reflects the experts’ judgement capacity and familiarity index. The reliability of the expert consultation was tested via four indicators [13]: the positive coefficient of experts; the coefficient of expert authority; Kendall’s W; the mean and coefficient of variation of the project importance assignment; and the judgement result of the item and the addition, deletion or modification of the item.

On the basis of the Delphi survey findings, the research team transformed the project repository into a predictive version of the scale, and the invited experts established the scoring system for the collection of the participants’ responses.

### Evaluation and exploration of the items

#### Cross-sectional study

We conducted a quantitative cross-sectional study.

##### Study subjects

A convenience sampling method was employed to select patients who were diagnosed with dry eye syndrome from the ophthalmology outpatient department of a tertiary Grade A hospital in Guangzhou between October 2024 and March 2025. The inclusion criteria for patients were as follows: (1) patients who met the diagnostic criteria for dry eye syndrome [14]: (i) presenting with at least one subjective symptom, such as ocular dryness, foreign body sensation, burning sensation, fatigue, discomfort, redness, or fluctuating vision; (ii) having an Ocular Surface Disease Index (OSDI) score of ≥ 13; and (iii) having a non-invasive break-up time (NIBUT) of < 10 s; (2) patients who voluntarily agreed to participate in the study and signed the informed consent form; and (3) patients aged 18 years or older. The exclusion criteria were as follows: (1) history of ocular trauma, infection, or non-dry eye-related ocular inflammation; (2) any surgery within the past 6 months or poorly controlled systemic diseases; (3) co-existing primary or secondary ocular diseases such as glaucoma or diabetic retinopathy; (4) use of topical immunosuppressants, steroids, and/or glucocorticoids within 6 months; (5) diagnosis of autoimmune diseases (e.g., rheumatoid diseases, systemic lupus erythematosus); (6) active ocular inflammatory diseases including acute conjunctivitis, keratitis, or uveitis; (7) any concomitant health problem or disability (including psychological disorders) that impairs daily activities.

The sample size was established based on the general rule of factor analysis [15], which recommends a minimum of five respondents for each item. The questionnaires were distributed to 450 patients diagnosed with dry eye syndrome (presenting with at least one subjective symptom, such as ocular dryness, foreign body sensation, burning sensation, fatigue, discomfort, redness, or fluctuating vision; having an Ocular Surface Disease Index (OSDI) score of ≥ 13; and having a non-invasive break-up time (NIBUT) of < 10 s). After excluding eight invalid responses, 442 valid questionnaires were analyzed, yielding an effective response rate of 98.2%. This sample size is adequate for the study.

The first phase of the study (Initial Scale Formation) did not involve participants. This sample (*n* = 422) was used in the second phase (Evaluation and exploration of the items) and the third phase (Psychometric Evaluation and Forming the Final Scale). In the third phase of factor analysis, according to the recommended sample size from general guidelines, the sample size for exploratory factor analysis (EFA) should be no fewer than 100 participants, while the sample size for confirmatory factor analysis (CFA) should exceed 200 [15, 16]samples. The participants were randomly assigned to two groups, one for EFA (*n* = 156) and the other for CFA (*n* = 266).

#### Items analysis

The items of the pretest scale were evaluated using a range of analytical methods, including a critical ratio analysis, correlation coefficient analysis, and internal consistency analysis. (a) The samples were divided into a high group (top 27% scores) and a low group (bottom 27% scores), and the two groups of samples were analysed to appraise the discrimination of the items. The acceptable critical ratio for each item was 3.0 or above and significant (*P* < 0.05) [17]. (b) Item-total correlation coefficients were calculated to assess the applicability of the items, with a minimum correlation coefficient of 0.3 [12]. (c) The homogeneity of the items was assessed using the Cronbach’s α coefficient after each item was deleted. Ideally, deleting any item should not increase the total Cronbach’s α coefficient. If the deletion of a particular item results in a higher Cronbach’s α coefficient, it indicates that the item adversely affects the scale’s internal consistency. Therefore, its removal should be considered. (d) Preliminary exploratory factor analysis was adopted to explore the factor loadings to evaluate the stability of the items. The recommended minimum factor loading was 0.4, and no cross-loadings were permitted. If any item failed to meet any of these conditions, it was excluded from the pretest scale [18, 19].

### Psychometric evaluation and forming the final scale

#### Reliability analysis

**Internal consistency reliability and split-half reliability**

To evaluate the internal consistency reliability of the scale, the Cronbach’s α coefficient and the split-half reliability coefficient were calculated. To establish good internal consistency and reliability, the Cronbach’s α coefficient should be 0.70 or higher. Split-half reliability refers to the correlation coefficient calculated between two halves of a scale after its completion or division by the odd-even method. A higher correlation coefficient indicates greater reliability [20].

**Test-retest reliability**

This study employed the intraclass correlation coefficient (ICC) to assess the stability of the scale. The ICC ranges from 0 to 1. Generally, an ICC greater than 0.7 indicates acceptable test-retest reliability of the scale [21]. In this part of the study, 30 patients with dry eye were selected for repeated measurements before and after a two-week interval, and the ICCs of the scale and the scores of each dimension from the two assessments were calculated.

#### Validity analysis

**Content validity**

The content validity of the scale was assessed by seven invited experts. The inclusion criteria for the experts were consistent with the inclusion criteria for the previous Delphi experts. A four-point Likert scoring system ranging from one point (irrelevant) to four points (very relevant) was used to collect expert responses. The content validity index of the item (I-CVI) was calculated as the ratio of the number of experts who ranked an item as 3 or 4 points to the total number of experts. For the content validity index of the scale (S-CVI), the average of all item I-CVI scores was calculated. To meet the criteria for content validity, an I-CVI score of 0.78 or above and an S-CVI score of 0.90 or higher were needed [22].

**Construct validity**

(1) EFA was employed to reveal the underlying factor structure and establish consistency with the conceptual framework. The suitability of the data for EFA was determined by Bartlett’s test of sphericity and the Kaiser-Meyer-Olkin (KMO) measure, with significant results (*P* < 0.05) and a KMO value exceeding 0.6 serving as prerequisites. All common factors should collectively account for more than 40% of the total variance [23–25]. The criteria for factor deletion were as follows: ① the eigenvalue of each factor exceeds 1; ② the maximum loading value is greater than 0.4; and ③ each common factor comprises at least three items [26].

(2) CFA was used to assess the degree of congruence between the measurement scale and the conceptual model [26]. To examine the structural validity of the scale, we conducted CFA based on previous findings. Fit indices were employed to reflect the degree of correspondence between the structure of the scale and theoretical concepts, and the goodness-of-fit and validity of the structural model were assessed. The primary indicators for evaluating the model-data fit included the following: ①Goodness-of-Fit Chi-Square Test (χ2): a *χ*^*2*^*/df* ratio < 3 indicates excellent model fit, whereas < 5 suggests acceptable fit; ②Comparative Fit Index (CFI) ranging from 0 to 1, with values closer to 1 indicating better fit and CFI > 0.90 generally considered satisfactory; ③a Tucker-Lewis Index (TLI) ranging from 0 to 1, with values approaching 1 indicating superior fit and TLI > 0.90 typically regarded as favourable; and ④Root Mean Square Error of Approximation (RMSEA), where RMSEA < 0.05 signifies excellent model-data fit and RMSEA < 0.08 indicates acceptable fit; and ⑤Standardized Root Mean-Square Residual (SRMR) < 0.8 considered indicative of accurate model fit.

### Data collection

During the initial phase, following a concise introduction that outlined the research objectives and significance, a compressed package containing informed consent forms and expert consultation questionnaires was disseminated to specialists via email. The experts were asked to provide feedback and recommendations within a two-week period after receipt of the survey instruments. During the subsequent phase, a questionnaire-based investigation was conducted with 422 patients who were diagnosed with dry eye syndrome. Prior to participation, all patients were thoroughly briefed on the purpose, importance, voluntary nature, and anonymity protocols of the study. Among the 450 patients invited to participate, 422 provided anonymous responses to the questionnaire after providing their consent. It is important to note that this study was conducted within a Chinese linguistic context. All questionnaires were administered in Chinese.

### Data analysis

The data were analysed using SPSS 26.0 and Mplus 8.0 software. The Delphi survey method was employed to revise the scale items and evaluate the content validity of the scale. For the EFA, the maximum variance rotation method was used to investigate the factor structure. Structural equation modelling (SEM) with the maximum likelihood method was applied to verify the consistency between the factor structure and the theoretical expectations. To determine the reliability of the scale, both internal consistency analysis and split-half reliability analysis were conducted to assess the homogeneity and stability of the scale.

### Ethical consideration

All procedures in this study were conducted in strict accordance with the Declaration of Helsinki (1964) and its subsequent amendments. Written informed consent was obtained from all participants. The participants were permitted to withdraw from the study at any time without the obligation to respond to any inquiries. The research protocol received ethical approval from the institutional review board of Nanfang Hospital, Southern Medical University (Approval No. NFEC-BPE-156).

## Results

### Initial scale development

#### Results of expert consultation

A total of 23 experts were selected for the consultation, including 2 nursing management experts, 3 clinical ophthalmology experts, 15 ophthalmic nursing experts, and 3 scale development experts. All 23 experts participated in two rounds of expert consultation. The participants’ age range was 32 to 57 years (mean ± SD: 43.39 ± 8.436 years), and their work experience ranged from 6 to 39 years (mean ± SD: 21 ± 10.569 years). Among them, 7 held senior professional titles, 8 held associate senior titles, and 8 held intermediate titles. With respect to academic qualifications, 4 held doctoral degrees, 8 held master’s degrees, and 11 held bachelor’s degrees.

During the two rounds of expert consultations, the effective response rate of experts reached 100%. In the first round, a total of 49 constructive suggestions were collected from 11 experts, while in the second round, 8 constructive suggestions were put forward by 3 experts. The proportions of experts providing suggestions in the two rounds were 48% and 13% respectively, indicating a high level of enthusiasm among the experts. The bases for experts’ judgments in the two rounds were 0.84 and 1.00 respectively. The degrees of experts’ familiarity with the content of the inquiry letters were 0.84 and 0.87 respectively. The expert authority coefficients (Cr) were 0.84 and 0.89 respectively, indicating that the results of this study are highly reliable [13]. The coefficient of variation of the importance score of the expert items in the first round of consultation ranged from 0.060 to 0.290, with a Kendalll’s W value of 0.203 (*χ*^2^ = 126.127, *df* = 27, *p* < 0.001). The coefficient of variation for the second round of expert consultation ranged from 0.000 to 0.111, with a Kendall’s W value of 0.075 (*χ*^2^ = 45.146, *df* = 26, *p* = 0.011).

In accordance with the screening criteria of the scale, after the first round of expert consultation, in combination with expert opinions and the brainstorming discussions of the working group, two dimensions and one item were added, one item was deleted, and the content and wording of eight items were revised. After the second round of expert consultation, in light of expert opinions and the brainstorming discussions of the working group, the content and wording of two items were modified. Lifestyle Adherence Assessment Scale for Patients with Dry Eye Syndrome (First draft) was formed with 6 dimensions and 27 items(Table 1).

**Table 1:** Initial version of the scale

Dimensions	Initial items
A Dietary Habit Adherence	A1 adheres to a daily dietary regimen comprising vegetables, fruits, fish, whole grains, olive oil, and foods rich in omega-3 fatty acids.
	A2 I will conscientiously limit my daily intake of high-fat foods, including fatty meats, animal offal, cream-based products, chocolate, and ice cream.
	A3 I seldom consume alcoholic beverages.
B Adherence to Electronic Screen Usage	B1 My daily cumulative usage duration of video display terminals, including mobile phones and computers, is less than 8 h.
	B2 I will consciously reduce the usage of electronic devices and allocate more time for rest.
	B3 After viewing a video display terminal for 20 min, I will focus on an object located at a distance of 6 m for 20 s.
C Environmental Management Adherence	C1 I often work or live in heated or air-conditioned environments (such as cabins, indoors, vehicles).*
	C2 In heated, cooled, or dehumidified environments, I use a humidifier to maintain optimal humidity levels.
	C3 In my professional engagement with outdoor activities, I consistently use sunglasses and protective equipment as essential safety measures.
	C4 I seldom engage in smoking.
	C5 Close acquaintances, family members, and colleagues in the immediate social circle exhibit minimal smoking behaviour.
	C6 My average daily sleep duration exceeds five hours.
	C7 I will implement non-pharmacological interventions to optimize sleep quality.
D Adherence to Ophthalmic Aesthetic Practices	D1 I wear contact lenses for less than 8 h daily.
	D2 I would opt for daily disposable contact lenses with superior wettability characteristics.
	D3 I meticulously cleanse my hands prior to handling contact lenses, strictly adhere to prescribed wearing durations, and maintain standardized care protocols for contact lens hygiene.
	D4 I endeavour to minimize the use of ocular cosmetics to the greatest extent possible.
	D5 I use certified and compliant eye makeup and makeup removal products and adhere to standardized protocols for cosmetic cleaning and storage.
	D6 I have a routine of undergoing various ophthalmic aesthetic procedures, including eyeliner tattooing, eyelash perming, eyelash tinting, false eyelash application, and the use of double eyelid tape. *
E Exercise Habit Adherence	E1 I regularly engage in blinking exercises as part of my routine.
	E2 After 20 min of continuous work or study, I make a conscious effort to rest with my eyes closed.
	E3 I engage in physical exercise at least three times per week, with each session lasting more than 30 min.
F Adherence to Follow-up Practices	F1 I independently purchase medication for self-treatment based on advertisements, recommendations from acquaintances, or other available channels*
	F2 I acquire knowledge related to dry eye syndrome through official channels, including hospital official accounts, educational materials, and health seminars.
	F3 Upon experiencing symptoms such as foreign body sensation, pain, and excessive tearing in the eyes, I proactively seek medical consultation.
	F4 I will discontinue the treatment independently upon perceiving an improvement in my symptoms*
	F5 I will undergo regular dry eye examinations as part of my routine healthcare protocol

Note: * For reverse-scored items

### The evaluation and exploration of the items

#### Sociodemographic characteristics of the participants

This study included 422 patients, 128 males and 294 females, who were diagnosed with dry eye syndrome. The mean age of the participants was 39.78 ± 14.379 years. A significant majority of the participants had attained higher education (72.3%), and the majority were married (60.9%). In terms of monthly income, 28.4% earned less than 5,000 RMB, whereas 38.6% reported a condition with a duration exceeding one year. The majority of the participants resided in urban areas (83.4%) (Table 2).

**Table 2:** Sociodemographic characteristics of the participants (*n* = 422)

Sociodemographic Characteristics		*n*(%)	
Gender	Male	128	30.3
	Female	294	69.7
Marital status	Single	159	37.7
	Married	257	60.9
	Divorced/separated	6	1.4
Education level	Primary education or lower	18	4.3
	Secondary education	99	23.5
	Higher education	305	72.3
Monthly income (RMB)	≤ 5000	120	28.4
	5000–10,000	120	28.4
	10,000–15,000	58	13.7
	>15,000	124	29.4
Residence	Village	70	16.6
	City	352	83.4
Duration of dry eye (months)	<3	176	41.7
	3–6	48	11.5
	6–12	35	8.3
	≥ 12	163	38.6
NIBUT (seconds)	OD	6.80(7.25,7.97)^a^	
	OS	7.35(7.31,8.36) ^a^	
OSDI (score)		31.25(34.19,37.55) ^a^	
TMH (millimeter)	OD	0.17(0.17,0.21)^a^	
	OS	0.17(0.01,1.19) ^a^	

Note: ^a^ Median and quartiles [M(P25, P75)]; *OSDI*: Ocular surface disease index; *NIBUT*: Non-Invasive Break-Up Time; *TMH*: Tear Meniscus Height

#### Results of item analysis

During the project analysis, the critical ratio of the project ranged from 1.138 to 10.518 (*P* < 0.05), and the critical ratios of B1, C1, F1, and F4 were less than 3.0. Pearson correlation results indicated that with the exception of C1 (*p* = 0.235), the differences in the remaining items were statistically significant (*P* < 0.001). However, items B1, C2, C6, D6, F1, and F4 exhibited low correlations (*r* < 0.3). The Cronbach’s α coefficient for the total scale was 0.652, but the deletion of items B1 (0.664), C1 (0.670), F1 (0.663), and F4 (0.660) resulted in an increase in the Cronbach’s α coefficient for the total scale and its dimensions, leading to their removal. Except for item C7 (0.293), the item-total correlations for all the items ranged between 0.442 and 0.857. Considering these results (Table 3), items B1, C1, D6, F1, and F4 were removed from the draft, resulting in a 22-item scale.

**Table 3:** Item analysis of the scale

Item	t Value	*P*	Factor loading	Cronbach’s α if item deleted	Item-total correlation coefficients	*P*	Retained item
A1	7.179	<0.001	0.596	0.638 (↓)	0.357	<0.001	√
A2	8.029	<0.001	0.654	0.640 (↓)	0.351	<0.001	√
A3	7.124	<0.001	0.715	0.647 (↓)	0.332	<0.001	√
B1	2.956	0.003	0.651	0.664 (↑)	0.155	0.001	×
B2	6.347	<0.001	0.74	0.639 (↓)	0.349	<0.001	√
B3	6.198	<0.001	0.525	0.643 (↓)	0.302	<0.001	√
C1	1.138	0.256	0.857	0.670 (↑)	0.058	0.235	×
C2	4.686	<0.001	0.562	0.645 (↓)	0.276	<0.001	√
C3	6.987	<0.001	0.685	0.637 (↓)	0.371	<0.001	√
C4	7.077	<0.001	0.727	0.643 (↓)	0.360	<0.001	√
C5	9.136	<0.001	0.735	0.638 (↓)	0.377	<0.001	√
C6	4.918	<0.001	0.541	0.646 (↓)	0.259	<0.001	√
C7	7.029	<0.001	0.293	0.642 (↓)	0.350	<0.001	√
D1	6.436	<0.001	0.785	0.636 (↓)	0.384	<0.001	√
D2	7.591	<0.001	0.846	0.630 (↓)	0.441	<0.001	√
D3	6.872	<0.001	0.674	0.632 (↓)	0.439	<0.001	√
D4	5.488	<0.001	0.642	0.642(↓)	0.343	<0.001	√
D5	8.302	<0.001	0.728	0.627 (↓)	0.484	<0.001	√
D6	3.447	0.001	0.442	0.650 (-)	0.229	<0.001	×
E1	5.228	<0.001	0.854	0.641 (↓)	0.320	<0.001	√
E2	5.832	<0.001	0.81	0.638 (↓)	0.359	<0.001	√
E3	6.522	<0.001	0.484	0.642 (↓)	0.335	<0.001	√
F1	2.639	0.009	0.672	0.663 (↑)	0.167	0.001	×
F2	10.518	<0.001	0.714	0.628 (↓)	0.456	<0.001	√
F3	7.580	<0.001	0.728	0.633 (↓)	0.423	<0.001	√
F4	2.862	0.005	0.628	0.660 (↑)	0.127	0.009	×
F5	5.471	<0.001	0.559	0.640 (↓)	0.342	<0.001	√

Note: ‘√’ indicates that the item was selected; ‘×’ indicates that the item was deleted. “↓” indicates that once the item is deleted, the Cronbach’s α decreases

### The psychometric evaluation of the scale

#### Reliability analysis

**Internal consistency reliability**

The Cronbach’s α coefficient of the scale was 0.709 (> 0.7), indicating good internal consistency of the scale. The split-half reliability coefficient of the scale was 0.559, suggesting a satisfactory level of reliability.

**Test-retest reliability**

A random sample of 30 patients from the formally surveyed cohort was selected for a retest reliability assessment of the scale after a two-week interval. The overall test-retest reliability coefficient of the scale was 0.931 (*P* < 0.001), indicating optimal reliability.

#### Validity analysis

**Content validity**

Seven experts who had not participated in the previous Delphi survey were invited to assess the content validity of the scale. As a result of the survey, the recovery rate of the consultation questionnaire was 1.000. The I-CVI was 1.000, and the S-CVI was 1.000 (Table 4).

**Table 4:** Content validity analysis for the developed scale

Item	Experts (score)							I-CVI	S-CVI
	1	2	3	4	5	6	7		
A1	4	4	4	4	4	4	4	1.000	1.000
A2	4	4	4	4	4	4	4	1.000	
A3	4	4	4	4	4	4	3	1.000	
B2	4	4	4	4	4	4	4	1.000	
B3	4	3	4	4	4	4	4	1.000	
C2	4	4	4	4	4	4	4	1.000	
C3	4	4	4	4	4	4	4	1.000	
C4	4	4	4	4	4	4	4	1.000	
C5	4	4	4	4	4	4	4	1.000	
C6	4	4	4	4	4	4	4	1.000	
C7	4	4	4	4	4	4	4	1.000	
D1	4	4	3	4	4	4	4	1.000	
D2	4	4	4	4	4	4	4	1.000	
D3	4	4	4	4	4	4	4	1.000	
D4	4	4	4	4	4	4	4	1.000	
D5	4	4	4	4	4	4	3	1.000	
E1	4	3	4	4	4	4	4	1.000	
E2	4	3	4	4	4	4	4	1.000	
E3	3	3	4	3	4	4	4	1.000	
F2	4	4	4	4	4	4	4	1.000	
F3	4	4	4	4	4	4	4	1.000	
F5	4	4	4	4	4	4	4	1.000	

**Construct validity**

#### Exploratory factor analysis

**Fig. 3:**
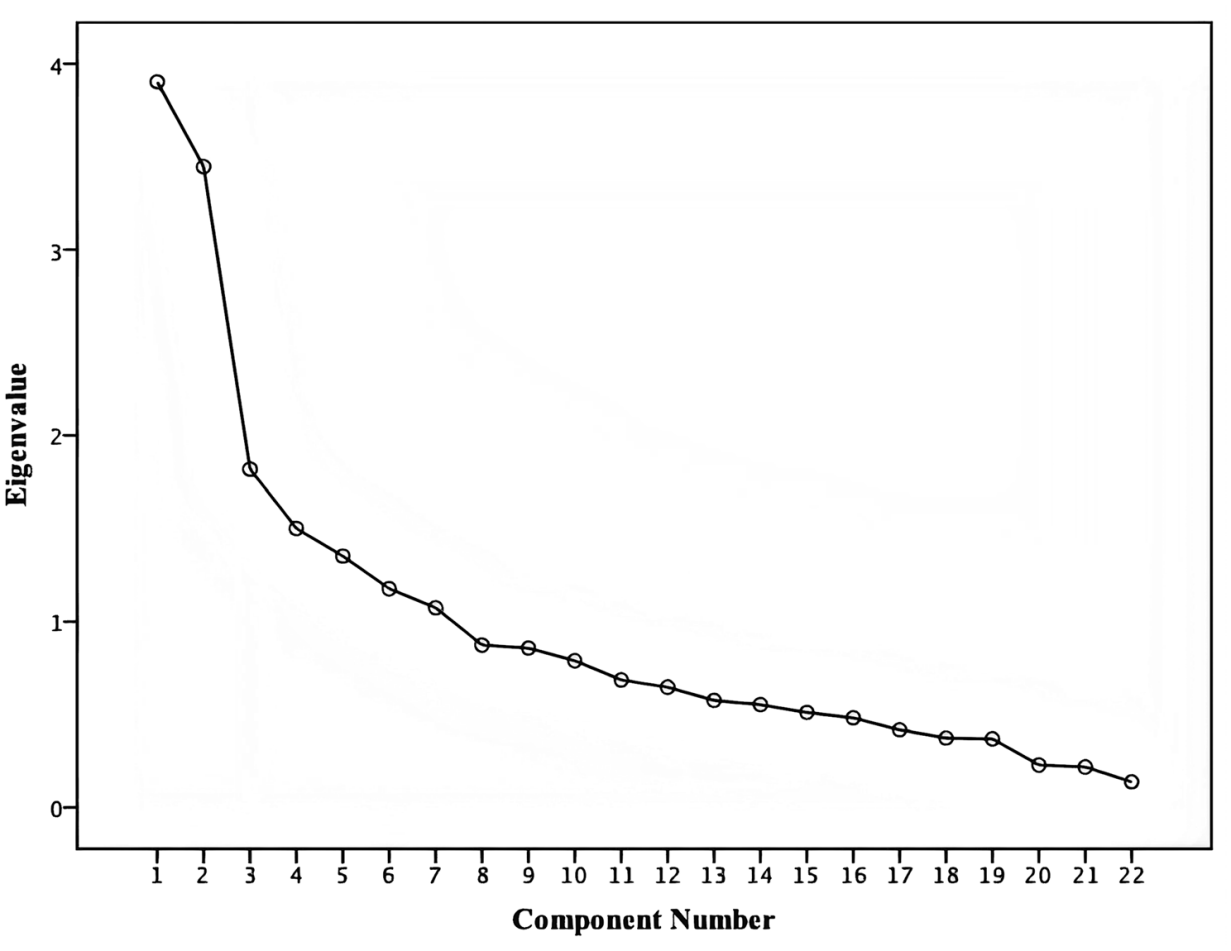
*Scree* plot of exploratory factor analysis for the Lifestyle Adherence Assessment Scale for Dry Eye Syndrome Patients

In this study, the KMO value of 0.712 and significant Bartlett’s test of sphericity (χ^2^ = 1111.098, *P* < 0.001) indicated that the data were suitable for EFA. Maximum variance rotation was performed to extract common factors, which resulted in five common factors with eigenvalues ≥ 1 that explained 54.648% of the total variation (Fig. 3). The factor loadings of these projects were all greater than 0.4. Among them, items A2, D3, and F5 with cross-loadings were deleted (Table 5). The resulting five-factor structure consisted of ophthalmic care standards, ocular strain regulation standards, seeking support, tobacco and alcohol consumption regulations and daily conduct guidelines, all of which are consistent with previous theoretical expectations.

**Table 5:** Pattern matrix of the scale after factor analysis (*n* = 156)

Item	Factor1	Factor2	Factor3	Factor4	Factor5
D2	0.811				
D5	0.805				
D1	0.777				
D4	0.748				
E2		0.817			
B3		0.769			
E1		0.761			
B2		0.678			
C7		0.514			
F2			0.694		
F3			0.668		
C3			0.558		
A3				0.774	
C5				0.745	
C4				0.682	
A1					0.639
E3					0.619
C6					0.541
C2					0.500

#### Confirmatory factor analysis

Based on the results of the EFA from the previous stage, CFA was conducted to further validate the structural validity of the scale. Item t19 (C2), which had a factor loading of -0.151, was considered for deletion. The original and revised model fit indices are presented in Table 6. The results indicated that *χ*^*2*^*/df* = 3.5143 < 5.00, *P* < 0.001, RMSEA = 0.037 (< 0.05), CFI = 0.956 (> 0.9), TLI = 0.946 (> 0.9), and SRMR = 0.073 (< 0.8). The selected fit indices demonstrated that the five-factor structure scale had a good fit and was relatively stable. The finalized scale is presented in Table 7.

**Table 6:** Confirmatory factor analysis model fitting values (*n* = 266)

Items	$\:{\boldsymbol{\chi\:}}^{2}$	df	$\:{\boldsymbol{\chi\:}}^{2}$/df	*P*	RMSEA	CFI	TLI	SRMR
Reference value			<5	<0.05	<0.1	>0.9	>0.9	<0.8
Initial value	600.939	171	3.5143	<0.001	0.037	0.956	0.946	0.073
Modified value	547.708	153	3.5798	<0.001	0.038	0.958	0.947	0.072

**Table 7:** Final version of the scale

Dimensions	Items
A Ophthalmic Care Standards	A1 I wear contact lenses for less than 8 h daily.
	A2 I would opt for daily disposable contact lenses with superior wettability characteristics.
	A3 I will endeavour to minimize the use of ocular cosmetics.
	A4 I will use certified and compliant eye makeup and makeup removal products, adhering to standardized procedures for cosmetic cleaning and storage.
B Ocular Strain Regulation Standards	B1 I will regularly engage in blink exercises to maintain optimal ocular health.
	B2 After engaging in continuous work or study for 20 min, I make a conscious effort to close my eyes and take a brief rest.
	B3 I will consciously reduce the use of electronic devices and allocate more time for rest.
	B4 After viewing a video display terminal for 20 min, I will focus on an object located at a distance of 6 m for 20 s. B5 I will implement non-pharmacological interventions to optimize sleep quality.
C Seeking supports	C1 I will acquire knowledge related to dry eye syndrome through official channels, including hospital official accounts, educational materials, and health seminars.
	C2 Upon experiencing symptoms such as foreign body sensation, ocular pain, and epiphora, I proactively seek medical consultation.
	C3 During my outdoor activities, I consistently use sunglasses and protective gear as essential safety measures.
D Tobacco and Alcohol Consumption Regulations	D1 I seldom consume alcoholic beverages.
	D2 I seldom engage in smoking.
	D3 The individuals in my immediate social circle, including close friends, family members, and colleagues, exhibit a notably low prevalence of smoking behaviour.
E Daily Conduct Guidelines	E1 I maintain a daily dietary regimen that includes a balanced intake of vegetables, fruits, fish, whole grains, olive oil, and foods rich in omega-3 fatty acids.
	E2 I engage in physical exercise for a minimum of three sessions per week, with each session lasting over 30 min.
	E3 My average daily sleep duration exceeds five hours.

## Discussion

Dry eye syndrome is a chronic ocular condition that currently lacks a definitive cure. Therapeutic interventions focus primarily on symptom management [4]. In cases of delayed or inadequate treatment, advanced stages of dry eye syndrome may manifest as corneal ulcers, perforation, secondary infections, and neovascularization, potentially leading to significant visual impairment and, in severe cases, permanent blindness. With prolonged progression of the disease, patients experience persistent ocular discomfort and irritation, which leads to a gradual increase in disease burden. This disease not only causes significant inconvenience in daily life but also imposes substantial psychological stress on patients [6, 27]. Research has shown that unhealthy lifestyle practices are significant risk factors for chronic diseases. Adopting a well-structured and scientifically validated lifestyle constitutes a crucial approach to controlling the progression of dry eye disease and mitigating associated complications. These behavioural factors encompass psychological elements, environmental management, and detrimental lifestyle habits, among others [5]. Current dry eye assessment tools, such as the OSDI, focus primarily on evaluating symptom severity and quality of life or the development of assessment tools for treatment adherence under other ophthalmic conditions [28–30]. Some scholars have independently developed a Dry Eye Patient Ocular Hygiene Adherence Scale to assess patients’ adherence to ocular hygiene practices. The scale comprises seven items, and each requires a binary response of “yes” or “no,” with a total score ranging from 0to 7. A score of 7 indicates excellent adherence, scores of 6 − 5 represent moderate adherence, and scores of 4 or below signify poor adherence. However, it should be noted that this scale has not undergone rigorous reliability and validity testing, and its feasibility and scientific rigor remain to be substantiated [31]. In summary, a comprehensive review of the literature revealed that there is currently no professionally developed and disease-specific scale for assessing lifestyle adherence in patients with dry eye syndrome. Existing studies rely predominantly on self-designed questionnaires, which have limitations in both measurement content and methodological rigor. The development of this scale will address this critical gap by providing a standardized adherence assessment tool for clinical practice, thereby facilitating a paradigm shift in dry eye management from “symptom control” to “behavioural intervention.”

This study involved a rigorous evidence-based review with stringent evidence screening, evaluation, extraction, and synthesis of high-quality evidence demonstrating the association between unhealthy lifestyle factors and dry eye syndrome [9]. Through panel discussions, the dimensions and items of the scale were preliminarily established. Through two rounds of Delphi surveys, a pretest version comprising six dimensions and 27 items was developed. The Delphi surveys demonstrated a 100% questionnaire return rate, with expert authority coefficients of 0.857 and 0.894, respectively. Kendall’s coefficient of concordance was 0.724, indicating satisfactory levels of enthusiasm, authority, and consistency among the experts regarding the scale.

In the item analysis, the critical ratios of items B1, C1, F1, and F4 were found to be less than 3.0, whereas the remaining items met the standard criteria, indicating that the scale items possessed a degree of discriminative validity. Correlation analysis revealed that with the exception of item C1 (*p* = 0.235), which was subsequently removed, all the other items demonstrated statistical significance (*P* < 0.001), suggesting inter-item associations within the scale. However, items B1, C2, C6, D6, F1, and F4 exhibited low correlations (*r* < 0.3). Furthermore, the removal of items B1, C1, F1, and F4 resulted in an increase in the Cronbach’s α coefficients for both the total scale and its dimensions. After comprehensive consideration of these metrics and subsequent group discussion, items B1, C1, D6, F1, and F4 were eliminated, resulting in the formation of a refined 22-item scale.

This study validated the internal consistency and test-retest reliability of the scale. The internal consistency reliability coefficient exceeded the recommended reference value at 0.709, whereas the split-half reliability reached 0.559, indicating favourable results and demonstrating good internal consistency of the evidence scale. Furthermore, the test-retest reliability coefficient was 0.931, confirming the scale’s satisfactory stability over time. In conclusion, the Lifestyle Adherence Assessment Scale for Dry Eye Patients has sound scientific validity and reliability.

After assessing the reliability of the measurement scale, we validated its content validity and construct validity. With respect to content validity, both the I-CVI and S-CVI exceeded the recommended threshold values [22], demonstrating the representativeness and applicability of the scale items. Construct validity, a crucial indicator for evaluating the stability of the scale, was initially examined through principal component analysis and varimax rotation, which extracted five common factors with a cumulative variance contribution rate of 54.648%. All item factor loadings exceeded 0.4; however, items A2, D3, and F5 exhibited cross-loadings and were subsequently removed after discussion. The final results indicated good construct validity. To ensure the model fit of the scale, CFA was employed and revealed that item C2 had insufficient factor loading and was considered for removal. After its exclusion, the analysis was repeated, and the five-factor model demonstrated that all the fit indices met the standard criteria, indicating a good fit between the collected data and the theoretical model of the scale. Through reliability and validity testing, we ultimately developed a scale comprising five dimensions and 18 items. Overall, the fit of the scale’s structural model sufficiently supported the results of the EFA, which reflected the ideal structural validity of the scale and achieved the objective of assessing patients’ adherence to lifestyle changes.

The core achievement of this study is the development of Lifestyle Adherence Assessment Scale for Dry Eye Patients. The distinctive value of this instrument lies in its integration of disparate lifestyle risk factors into a structured and quantifiable framework, offering a clear clinical pathway from risk screening to targeted intervention. Unlike previous studies that often examined risk factors in isolation, this scale synthesizes five key dimensions—Ophthalmic Care Standards, Ocular Strain Regulation Standards, Seeking supports, Tobacco and Alcohol Consumption Regulations and Daily Conduct Guidelines—enabling clinicians to efficiently identify dominant risk profiles and deliver personalized guidance.

In the Ophthalmic Care Standards dimension, scores not only reflect usage frequency but also prompt detailed discussion of wear habits, makeup hygiene, and lens material selection (e.g., opting for moderate-water-content lenses), facilitating precise patient-specific recommendations [32–34]. The Ocular Strain Regulation Standards dimension translates scores into actionable behavioral plans; for example, patients with high digital exposure may be prescribed strict adherence to the 20-20-20 rule and structured blink-training regimens [35, 36]. Similarly, the Tobacco and Alcohol Consumption Regulations dimension provides an objective basis for delivering clear cessation advice [37].

Critically, the Daily Conduct Guidelines dimension moves beyond siloed advice by promoting an integrated approach—combining Mediterranean-style nutrition, regular physical activity (≥ 3 times/week), and adequate sleep (> 5 h/night)—to systemically improve metabolic health and ocular surface homeostasis [9, 38–40]. This holistic perspective represents a significant advance over fragmented lifestyle assessments.

Finally, the scale closes the loop through the help-Seeking supports dimension, encouraging patients to seek timely professional support (e.g., for lid hygiene protocols) and adopt protective measures (e.g., UV protection for outdoor workers) when high-risk scores or symptoms are detected [41]. Thus, the scale functions not only as an assessment tool but also as a clinical communication framework that supports shared decision-making. By translating generalized recommendations into individualized, trackable action plans, it significantly enhances the practicality, adherence, and effectiveness of lifestyle interventions in dry eye management.

## Limitations

This study has several limitations. First, the findings are situated within China’s specific socio-cultural context, as all instruments and communications were in Chinese; this may limit their cross-cultural generalizability. Second, the sample was drawn from a single tertiary hospital and consisted predominantly of highly educated patients (72.3%), which may restrict the applicability of the results to more diverse populations. Third, despite a factor loading below 0.40 in the confirmatory factor analysis, item C3 (from the support-seeking dimension) was retained due to its deemed clinical relevance. Finally, in striving for psychometric robustness, the scale development process may have excluded some relevant items, potentially affecting its comprehensiveness. Future research should focus on cross-cultural validation of the scale in diverse populations, its refinement with stricter quality control, and more in-depth investigations into the lifestyle factors of dry eye patients.

## Conclusions

In this study, we developed and validated a Lifestyle Adherence Assessment Scale for Patients with Dry Eye Syndrome. The 18-item scale, comprising five dimensions, demonstrated good psychometric properties, providing a reliable tool to guide clinical management and inform future interventions for DED patients. Future studies should test this scale in different populations and settings to confirm generalizability.

## Data Availability

The datasets used and/or analysed during the current study are available from the corresponding author on reasonable request.

## Abbreviations

I-CVI: Content validity index of the item
S-CVI: Content validity index of the scale
EFA: Exploratory factor analysis
CFA: Kaiser-Meyer-Olkin
CFA: Confirmatory factor analysis
CR: Composite reliability
OSDI: Ocular surface disease index
NIBUT: Non-invasive brup time
